# Initial low-dose computed tomography screening results and summary of participant characteristics: based on the latest Chinese guideline

**DOI:** 10.3389/fonc.2023.1085434

**Published:** 2023-05-24

**Authors:** Zixuan Zhang, Yinyan Gao, Shaohui Liu, Binrong Ding, Xuewei Zhang, Irene X. Y. Wu

**Affiliations:** ^1^ Xiangya School of Public Health, Central South University, Changsha, China; ^2^ Health Management Center, Xiangya Hospital, Central South University, Changsha, China; ^3^ Department of Geriatrics, The Third Xiangya Hospital, Central South University, Changsha, China; ^4^ Centre for Medical Genetics & Hunan Key Laboratory of Medical Genetics, School of Life Sciences, Central South University, Changsha, China; ^5^ National Clinical Research Center for Geriatric Disorders, Xiangya Hospital, Central South University, Changsha, China; ^6^ Hunan Provincial Key Laboratory of Clinical Epidemiology, Changsha, China

**Keywords:** lung cancer, screening, low-dose computed tomography, non-smokers, characteristics

## Abstract

**Background:**

Low-dose computed tomography (LDCT) has been promoted as a promising screening strategy for early detection of lung cancer. China released the latest lung cancer screening guideline in 2021. The compliance of the individuals who received LDCT for lung cancer screening with the guideline is unknown yet. It is necessary to summarize the distribution of guideline-defined lung cancer–related risk factors in the Chinese population so as to inform the selection of target population for the future lung cancer screening.

**Methods:**

A single-center, cross-sectional study design was adopted. All participants were individuals who underwent LDCT at a tertiary teaching hospital in Hunan, China, between 1 January and 31 December 2021. LDCT results were derived along with guideline-based characteristics for descriptive analysis.

**Results:**

A total of 5,486 participants were included. Over one-quarter (1,426, 26.0%) of the participants who received screening did not meet the guideline-defined high-risk population, even among non-smokers (36.4%). Most of the participants (4,622, 84.3%) were found to have lung nodules, while no clinical intervention was required basically. The detection rate of positive nodules varied from 46.8% to 71.2% when using different cut-off values for positive nodules. Among non-smoking women, ground glass opacity appeared to be more significantly common compared with non-smoking men (26.7% vs. 21.8%).

**Conclusion:**

Over one-quarter of individuals who received LDCT screening did not meet the guideline-defined high-risk populations. Appropriate cut-off values for positive nodules need to be continuously explored. More precise and localized criteria for high-risk individuals are needed, especially for non-smoking women.

## Graphical Abstract

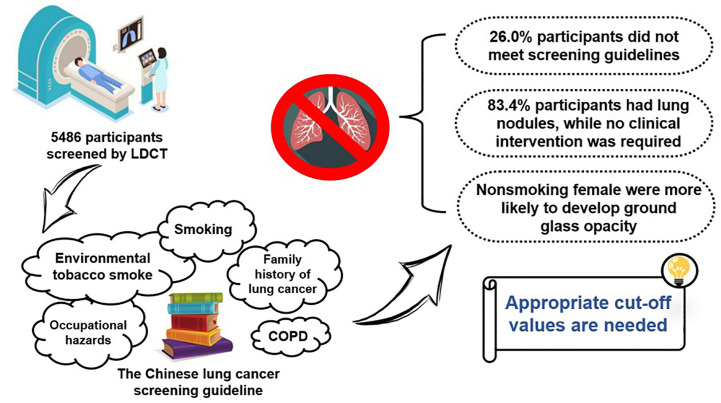

## Introduction

As one of the most serious cancers, lung cancer–related deaths accounted for 18.4% of global cancer-related deaths in 2018 (1). Since 2000, the total number of lung cancer incidence and mortality among Chinese residents has been on the rise (2), accounting for 37.0% and 39.8% of the global cases, respectively (3). Due to a poor prognosis (4) and the lack of effective clinical treatment options (5), early detection of lung cancer by screening with low-dose computed tomography (LDCT) to detect potentially malignant lung nodules (6) remains the primary strategy for long-term mortality reduction (7). Compared with conventional CT, LDCT scans have less radiation dose while ensuring higher image quality at the same time (8).

Theoretically, accurately identify all the high-risk individuals by screening will avoid 88% death from lung cancer (9). However, attention is needed to be paid for the side effects caused by screening, such as overdiagnosis (10) and radiation exposure (11). Hence, accurately identifying the target population is critical for developing a lung cancer screening strategy (12). Many countries or institutions have introduced different LDCT screening guidelines (13, 14), containing different definitions of high-risk individuals. The lung cancer screening guidelines in Western countries defined the high-risk individuals only based on age and smoking status (13), which were also accepted in the previous Chinese guidelines (14). In 2021, the latest Chinese guideline (15) *China guideline for the screening and early detection of lung cancer (2021, Beijing)* has been updated, adding extra risk factors such as passive smoking history, a family history of lung cancer, and hazardous occupational history for defining high-risk individuals. Although the recommended population for LDCT has been expanded, it is unclear how well the population currently screened for LDCT meets the definition of high-risk individuals. Lung cancer screening has not been popularized to the whole population in China. In most cases, residents receive LDCT screening at their own discretion during physical examination without considering eligibility and necessity. Therefore, it is necessary to summarize the relevant characteristics of the above-mentioned populations and inform where improvements are needed in LDCT screening for lung cancer at a hospital setting. Incompliance to the screening guideline may exacerbate the adverse effects of screening, which might also affect the allocation of medical resources (16).

Some studies (17, 18) have described the characteristics of the participants receiving the LDCT screening and the screening results based on the Western populations, with limited information from Asian population (19, 20), especially Chinese residents. A review (19) manifested that as of February 2019, there were six cohort studies that described the characteristics as well as LDCT results of the Chinese population (two studies from Taiwan). However, the above-mentioned studies, including the latest study that was conducted in 2018 (21), only focused on smokers, with very limited data on risk factors other than smoking. In the light of the uniqueness of lung cancer pathogenesis and pathological features in Asians (22), especially a higher proportion of non-smoking women (23), studies that provided information on Asian population, especially on non-smoking women, are needed. Furthermore, in addition to smoking status, other risk factors (e.g., passive smoking and a family history of lung cancer) (24) have been well documented to be associated with lung cancer. The distributions of these risk factors in the individuals receiving LDCT screening as well as the lung nodule population have not been well explored. It is necessary to sort out the screening results and to summarize the characteristics of different nodule types. A comprehensive summary of the initial LDCT screening results (e.g., the type and size of nodules detected) and the guideline-defined risk factors of the participants is needed to inform future lung cancer screening decisions and provide some hints for the follow-up management of the screening-positive population (20).

To sum up, based on the latest LDCT screening guideline in China and using the physical examination data from a tertiary teaching hospital in Hunan, China, *the present study aimed to (1) assess the compliance of individuals who received LDCT screening with the guideline (to explore the concordance between individuals who received LDCT screening and the guideline-defined screening population) and (2) summarize and compare the LDCT screening results (the presence, size, and type of lung nodules) of individuals with different smoking status.*

## Methods

### Study design

We adopted a cross-sectional study design using the *China guideline for the screening and early detection of lung cancer (2021, Beijing) (* 15 *)* as a reference to investigate the compliance of the participants with the guidelines and their initial screening results.

All the participants’ characteristics and LDCT screening results were based on the de-identified data exported from the hospital data management system. This study was approved by the Ethics Committee of Xiangya School of Public Health, Central South University (XYGW-2021-104).

### Study participants

We included all participants who underwent LDCT screening in the Health Management Center of Xiangya Hospital between 1 January and 31 December 2021. Our exclusion criteria were (1) participants with lung cancer within 5 years (25), *who generally received LDCT screening to assess the progress of lung cancer, or (2) participants with malignancy that has metastasized to the lungs, or (3) participants who did not complete the pre-examination questionnaire, which provided essential data on the interest characteristics (e.g., smoking status and a family history of lung cancer) of this study.*

### The criteria of high-risk individuals in the guideline

According to the *China guideline for the screening and early detection of lung cancer (2021, Beijing) (* 15 *)*, individuals with one or more of the following conditions are considered as high risk and are recommended to receive lung cancer screening: (1) age > 50 years; (2) smoking (smoking ≥ 30 packs/year); (3) passive smoking history; (4) with chronic obstructive pulmonary disease; (5) hazardous occupation exposure (≥1 year); and (6) having a first-degree relative diagnosed with lung cancer.

### Collection of guideline-based characteristics

All above-mentioned guideline-based characteristics were collected through the hospital pre-examination questionnaire system from 1 January to 31 December 2021. However, we were not able to collect data on chronic obstructive pulmonary disease as it was not covered in the questionnaire. The pre-examination questionnaire system was designed to collect background information (including demographic characteristics, previous health conditions, and family disease history) of the participants with a self-administrative questionnaire before undergoing any medical examination.

Specifically, participants were asked to provide the following information: (1) age and sex; (2) smoking status (divided into non-smokers, smokers, or ex-smokers); (3) passive smoking; (4) a family history of lung cancer (limited to first-degree relatives); and (5) hazardous occupation exposure (≥1 year). Of these, non-smokers (26) were defined as “never smoked or had smoked fewer than 100 cigarettes lifetime”; passive smoking (27) was defined as “inhaled smoke exhaled by smokers for at least 15 minutes a day on more than 1 day in a week.” The most complete questionnaire was selected when more than one version was identified for the same participant, with the remaining being used as supplementary. Participants were considered to be following the guidelines as long as they possessed any of these characteristics.

### Low-dose computed tomography screening results

All LDCT screening results were from the radiologist’s CT reporting system. Relevant LDCT screening results included (1) whether or not the LDCT scan was performed for the first time; (2) whether there was a lung disease (including tuberculosis, pneumonia/bronchitis, pulmonary bullae, emphysema, and atelectasis. Determined by radiographical findings and patient self-reports); (3) whether there was a lung nodule; and (4) positive nodules. When participants had lung nodules, their associated nodule characteristics were also recorded. Nodule-associated characteristics included (1) the number of nodules; (2) classification; (3) size; (4) type (e.g., solid, partial solid, ground glass opacity, and calcified nodule); and (5) whether the nodule borderline was clear. As there is no standardized criteria for positive nodules, we selected two criteria: non-calcified nodules ≥4 mm recommended in the National Lung Screening Trial (NLST) (28) and nodules ≥5 mm recommended based on the LDCT screening data of Shanghai residents in China (29). Nodule size was measured by the length and diameter, and the classification criteria referred to The Lung Reporting and Data System (Lung-RADS) categories (30). Specifically, the Lung-RADS 2 is defined as “Nonsolid nodules <30 mm or solid nodules <6 mm on baseline screening.” The Lung-RADS 3 is defined as “Nonsolid nodules ≥30 mm or solid nodules ≥6 to 8 mm at baseline.” The Lung-RADS 4 is defined as “Solid nodules≥8mm or part solid nodules ≥6mm.” Extensive details can be obtained in *Lung‐RADS Version 1.1.* Characteristics of the dominant nodule, which was considered to be most likely to receive treatment, were recorded when multiple nodules were detected (25).

When there was ambiguity about the results, the original CT image was checked. When multiple LDCT screening results for the same participant were found, the initial one was selected. The LDCT parameters used were as follows: 1.3 mm slice thickness, 1 mm slice spacing, 100 kV tube voltage, and 40–100 mA tube current.

### Data collection and collation

All the guideline-based characteristics and LDCT screening results were imported into Excel, with duplicates being eliminated. These data were then imported into Stata version 16.0 (Stata Inc., USA), using the “merge” command to identify the overlap between the two databases by matching age, sex, and medical number (a special number assigned by the hospital to each participant). A data set of LDCT screening results matching guideline-based characteristics was obtained for the further analysis.

### Statistical analysis

Given that smoking status is an important factor in lung cancer (31), the participants with both LDCT screening results and guideline-based characteristics were divided into three groups (non-smokers, smokers, and ex-smokers) to present the data. Taking into account that non-smokers deserve more attention in screening (32), their guideline-based characteristics and LDCT screening results were further grouped by sex (33) and presented. In addition, participants based on Lung-RADS 4 were most likely to develop lung cancer (30), and their relevant guideline-based characteristics were also presented separately to show differences between sexes.

The continuous variable (age) was described with mean and standard deviation, while categorical variables (e.g., sex and Lung-RADS category) were described with frequency and percentages. Differences among smoking groups were compared using the analysis of variance (ANOVA) for the continuous variable (age) and with the Pearson chi-square test and Fisher’s exact test for the categorical variables. If appropriate, the least significant difference-t (LSD-t) test and the partitions of the chi-square method were used for further pairwise comparisons. Similarly, differences between women and men in non-smokers and Lung-RADS 4 participants were compared by the Student’s t-test, Pearson chi-square test, and Fisher’s exact test where appropriated. *P*-values less than 0.05 were considered statistically significant. All the statistical analysis were performed with the SPSS-IBM, version 24.0 (SPSS Inc., USA).

## Result

### Data collection and collation

**Figure 1:**
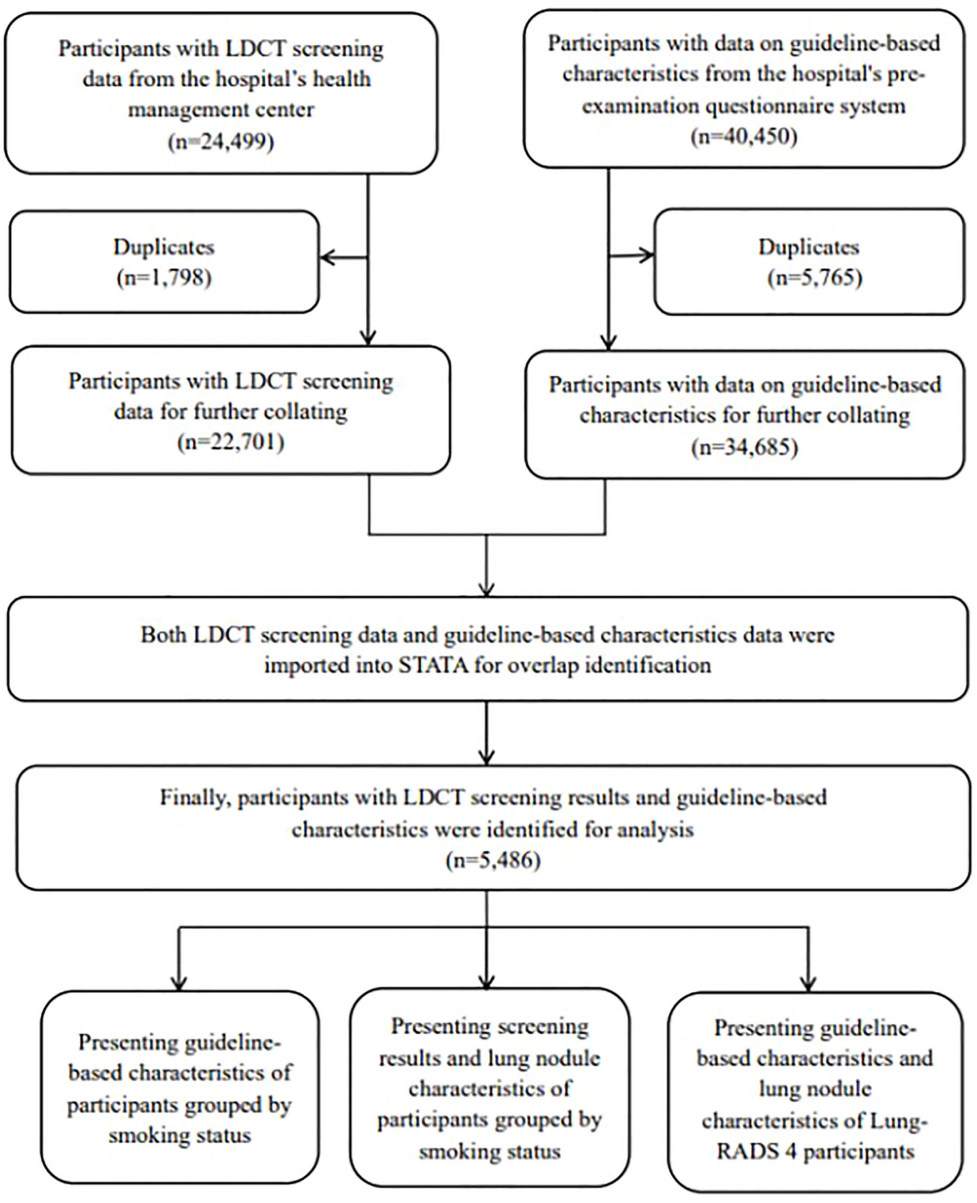
The process of data collection and collation.

We exported all the 24,499 LDCT screening records from 1 January 2021 to 31 December 2021. Participants with guideline-based characteristics (*n* = 40,450) during the same period were collected from the pre-examination system. Finally, participants with both LDCT screening results and guideline-based characteristics were successfully matched and included in the data analysis (*n* = 5,486) (**Figure 1**).

### Guideline-based characteristics

Among the 5,486 participants, non-smokers accounted for the majority (3,670, 66.9%), followed by smokers (1,514, 27.6%), and ex-smokers (302, 5.5%). Although the sex distribution of men and women was similar overall (53.8% vs. 46.2%), women dominated in non-smokers (66.8%) and men dominated in smokers (95.5%) and ex-smokers (95.0%). The age of the included participants ranged from 15 to 83 years (mean: 48.3 years, standard deviation: 10.4 years) with more than half under 50 years (3,132, 56.9%). Around one-fifth (768, 19.3%) had a history of passive smoking, while it is worth noting that more than half (59.0%) of the participants did not provide related information. Only a few participants had a family history of lung cancer (313, 5.7%) and hazardous occupation (403, 7.3%). Overall, most participants (4,060, 74.0%) met at least one guideline-based characteristic, while nearly a quarter of participants did not. When looking specifically at different smoking status groups, non-smokers seem to have worse compliance with the guidelines than ex-smokers (63.6% vs. 74.2%) (**Table 1**).

**Table 1:** Guideline-based characteristics of participants received Low-dose computed tomography (LDCT) screening^α^.

Characteristics	Total (N=5486)	Non-smokers^β^ (N=3670)	Smokers (N=1514)	Ex-smokers (N=302)		*p* value
Sex						<0.001^γ^
Male	2953 (53.8)	1220 (33.2)	1446 (95.5)	287 (95.0)		
Female	2533 (46.2)	2450 (66.8)	68 (4.5)	15 (5.0)		
Age range (years)						
≤30	256 (4.6)	131 (3.6)	114 (7.5)	11 (3.6)		
31-40	1118 (20.4)	626 (17.1)	426 (28.1)	66 (21.9)		
41-50	1749 (31.9)	1220 (33.2)	444 (29.3)	85 (28.1)		
51-60	1842 (33.6)	1327 (36.2)	419 (27.7)	96 (31.8)		
61-70	433 (7.9)	302 (8.2)	93 (6.2)	38 (12.6)		
≥71	88 (1.6)	64 (1.7)	18 (1.2)	6 (2.0)		
**Mean age ± SD** ^δ^ **(years)**	48.3±10.4	48.8±10.0	45.5±10.8	49.2±10.5		<0.001^ϵ^
Passive smoking history ^ζ^						<0.001^η^
Yes	768 (19.3)	670 (18.2)	–	98 (32.5)		
No	863 (21.7)	803 (21.9)	–	60 (19.8)		
Unknown	2341 (59.0)	2197 (59.9)	–	144 (47.7)	
Family history of lung cancer ^θ^						0.673
Yes	313 (5.7)	214 (5.8)	82 (5.4)	17 (5.6)		
No	4557 (83.1)	3030 (82.6)	1271 (84.0)	256 (84.8)		
Unknown	616 (11.2)	426 (11.6)	161 (10.6)	29 (9.6)		
Hazardous occupation ^μ^						<0.001^γ^
Building decoration	155 (2.8)	63 (1.7)	78 (5.2)	14 (4.6)		
Metal smelting	137 (2.5)	64 (1.7)	58 (3.8)	15 (5.0)		
Cook	96 (1.7)	58 (1.6)	34 (2.2)	4 (1.3)		
Related to pesticide exposure	15 (0.3)	11 (0.3)	4 (0.3)	0 (0.0)		
None	5083 (92.7)	3474 (94.7)	1340 (88.5)	269 (89.1)		
Guideline compliance						<0.001^η^
Yes	4060 (74.0)	2322 (63.6)	–	224 (74.2)		
No	1426 (26.0)	1348 (36.7)	–	78 (25.8)		

^α^The values are numbers (percentages), unless stated otherwise.

^β^Non-smokers were defined as never smoked or had smoked fewer than 100 cigarettes in their lifetime.

^γ^Statistical differences were found between the non-smokers and smokers, non-smokers and ex-smokers.

^δ^SD means Standard Deviation.

^ϵ^Statistical differences were found between the smokers and non-smokers, smokers and ex-smokers.

^ζ^Passive smoking history only considered in non-smokers and ex-smokers.

^η^Statistical difference was found between the non-smokers and ex-smokers.

^θ^Family history limited to first-degree relatives.

^μ^Exposed to occupational hazardous substances for one year or more.

### Low-dose computed tomography screening results

More than half of the participants (3,823, 69.7%) underwent LDCT scans for the first time. The vast majority (4,511, 82.3%) were lung disease–free, with the highest proportion being seen in non-smokers (86.8%), followed by ex-smokers (77.2%) and smokers (72.2%). Most of the participants (4,622, 84.3%) were found to have lung nodules of varying degrees, regardless of the smoking status (non-smokers: 84.5%, smokers: 83.8%, and ex-smokers: 83.8%). Different definitions of positive nodules have different detection rates, with the cut-off value of the NLST showing a much higher detection rate of positive nodules (71.2%) than that of the cut-off value recommended by the study in Shanghai (46.8%) (**Table 2**).

**Table 2:** Low-dose computed tomography (LDCT) screened results^α^.

Results	Total (N=5486)	Non-smokers^β^ (N=3670)	Smokers (N=1514)	Ex-smokers^β^ (N=302)	*p* value
First lung LDCT scan					<0.001^ν^
Yes	3823 (69.7)	2478 (67.5)	1131 (74.7)	214 (70.9)	
No	1663 (30.3)	1192 (32.5)	383 (25.3)	88 (29.1)	
Lung disease ^ο^					<0.001^ν^
Tuberculosis	103 (1.9)	63 (1.7)	35 (2.3)	5 (1.7)	
Pneumonia/ Bronchitis	503 (9.2)	252 (6.9)	214 (14.1)	37 (12.3)	
Pulmonary bullae	343 (6.3)	146 (4.0)	171 (11.3)	26 (8.6)	
Emphysema	292 (5.3)	77 (2.1)	188 (12.4)	27 (8.9)	
Atelectasis	15 (0.3)	11 (0.3)	3 (0.2)	1 (0.3)	
None	4511 (82.3)	3185 (86.8)	1093 (72.2)	233 (77.2)	
Lung nodules					0.778
Yes	4622 (84.3)	3101 (84.5)	1268 (83.8)	253 (83.8)	
No	864 (15.7)	569 (15.5)	246 (16.2)	49 (16.2)	
Positive nodules^Σ^					
NLST definition^ψ^	3908 (71.2)	2617 (71.3)	1076 (71.1)	215 (70.0)	0.887
Shanghai definition^ω^	2565 (46.8)	1697 (46.2)	721 (47.6)	147 (48.7)	0.609

^α^ The values are numbers (percentages), unless stated otherwise.

^β^ Non-smokers were defined as never smoked or had smoked fewer than 100 cigarettes in their lifetime.

^ν^ Statistical difference was found between the non-smokers and smokers.

^ο^ Due to the fact that a participant might have multiple lung diseases, the total percentage was over one hundred.

^Σ^ The number of positive nodules detected by different definitions and the corresponding percentage.

^ψ^ Non-calcified nodules ≥4 mm.

^ω^ Nodules ≥5 mm.

### Characteristics of the detected lung nodules

Among the 4,622 participants who have been detected with lung nodules, the majority of them (2,978, 64.4%) had four or more nodules. Most nodules were categorized as Lung-RADS 2 (3,414, 73.9%), followed with Lung-RADS 3 (1,133, 24.5%), while Lung-RADS 4 (75, 1.6%) accounted for the least. The predominance of four or more nodules and Lung-RADS 2 were also seen among participants with different smoking status. The detected lung nodules were generally smaller than 10.0 mm (<5.0 mm: 44.5%, 5.0–9.9 mm: 51.4%), while very few nodules had a size larger than 20.0 mm (20.0–29.9 mm: 0.3%, ≥30 mm: 0.1%). As for the types of the nodule, solid nodules accounted for approximately half (50.3%), followed by ground glass opacity (23.4%), subsolid nodules (7.6%), and calcified nodules (0.8%), while the remaining 17.9% were classified as unclear. Smokers showed a statistically significant lower proportion of solid nodules compared to that of non-smokers (54.2% vs. 48.1%) while having a higher proportion of ground glass opacity (25.1% vs. 19.7%) (**Table 3**).

**Table 3:** Characteristics of detected lung nodules^α^.

Characteristics^π^	Total (N=4622)	Non-smokers^β^ (N=3101)	Smokers (N=1268)	Ex-smokers (N=253)	*p* value
Nodule number					0.282
1	842 (18.2)	569 (18.3)	217 (17.1)	56 (22.1)	
2	587(12.7)	399 (12.9)	160 (12.6)	28 (11.1)	
3	215 (4.7)	152 (4.9)	57 (4.5)	6 (2.4)	
≥ 4	2978 (64.4)	1981 (63.9)	834 (65.8)	163 (64.4)	
Lung-RADS^ρ^ category					0.445
Lung-RADS 2	3414 (73.9)	2290 (73.8)	946 (74.6)	178 (70.3)	
Lung-RADS 3	1133 (24.5)	759 (24.5)	305 (24.1)	69 (27.3)	
Lung-RADS 4	75 (1.6)	52 (1.7)	17 (1.3)	6 (2.4)	
Nodule size ^σ^ (mm)					0.764
< 5.0	2057 (44.5)	1404 (45.3)	547 (43.1)	106 (41.9)	
5.0-9.9	2377 (51.4)	1578 (50.9)	664 (52.4)	135 (53.3)	
10.0-19.9	169 (3.7)	107 (3.4)	52 (4.1)	10 (4.0)	
20.0-29.9	13 (0.3)	9 (0.3)	3 (0.2)	1 (0.4)	
≥ 30.0	6 (0.1)	3 (0.1)	2 (0.2)	1 (0.4)	
Nodule type					<0.001^ν^
Solid	2325 (50.3)	1493 (48.1)	687 (54.2)	145 (57.3)	
Partial solid	352 (7.6)	254 (8.2)	79 (6.2)	19 (7.5)	
Ground glass opacity	1081 (23.4)	778 (25.1)	250 (19.7)	53 (21.0)	
Calcified nodule	38 (0.8)	24 (0.7)	14 (1.1)	0 (0.0)	
Unclear	826 (17.9)	552 (17.9)	238 (18.8)	36 (14.2)	
Nodule borderline					0.509
Regular	244 (5.3)	158 (5.1)	68 (5.4)	18 (7.1)	
Irregular	103 (2.2)	74 (2.4)	23 (1.8)	6 (2.4)	
Unclear	4275 (92.5)	2869 (92.5)	1177 (92.8)	229 (90.5)	

^α^ The values are numbers (percentages), unless stated otherwise.

^π^ One dominant nodule was selected if multiple nodules were found.

^β^ Non-smokers were defined as never smoked or had smoked fewer than 100 cigarettes in their lifetime.

^ρ^ Lung-RADS means Lung Imaging Reporting and Data System.

^σ^ Based on the largest diameter of the nodule.

^ν^ Statistical difference was found in solid and ground glass opacity between the non-smokers and smokers.

Among the 3,101 non-smokers who have been detected with lung nodules, most nodule characteristics distribution of women (*n* = 2,074) and men (*n* = 1,027) tended to be consistent without statistical significance. Concretely, four or more nodules (women: 64.5%; men: 62.6%) and Lung-RADS 2 (women: 73.5%; men: 74.7%) were most common. The nodule size was mainly concentrated in the two ranges of <5 mm (women: 46.0%; men: 43.8%) and 5–9.9 mm (women: 50.2%; men: 52.3%). Notably, non-smoking women had a statistically significant lower proportion of solid nodules (45.5% vs. 53.5%) and a higher proportion of ground glass opacity (26.7% vs, 21.8%) compared with non-smoking men (**Table 4**).

**Table 4:** Characteristics of detected lung nodules among non-smokers (N=3101) ^α^.

Characteristics^π^	Female (N=2074)	Male (N=1027)	*p* value
Nodule number			0.088
1	383 (18.5)	186 (18.1)	
2	246 (11.9)	153 (14.9)	
3	107 (5.1)	45 (4.4)	
≥ 4	1338 (64.5)	643 (62.6)	
Lung-RADS ^ρ^ category			0.826
Lung-RADS 2	1523 (73.5)	767 (74.7)	
Lung-RADS 3	515 (24.8)	244 (23.8)	
Lung-RADS 4	36 (1.7)	16 (1.5)	
Nodule size ^σ^ (mm)			0.469
< 5	954 (46.0)	450 (43.8)	
5-9.9	1041 (50.2)	537 (52.3)	
10-19.9	69 (3.3)	38 (3.7)	
20-29.9	8 (0.4)	1 (0.1)	
≥ 30	2 (0.1)	1 (0.1)	
Nodule type			0.001^ν^
Solid	944 (45.5)	549 (53.5)	
Partial solid	181 (8.7)	73 (7.1)	
Ground glass opacity	554 (26.7)	224 (21.8)	
Calcified nodule	18 (0.9)	6 (0.6)	
Unclear	377 (18.2)	175 (17.0)	
Nodule borderline			0.545
Regular	112 (5.4)	46 (4.5)	
Irregular	49 (2.4)	25 (2.4)	
Unclear	1913 (92.2)	956 (93.1)	

^α^ The values are numbers (percentages), unless stated otherwise.

^π^ One dominant nodule was selected if multiple nodules were found.

^ρ^ Lung-RADS means Lung Imaging Reporting and Data System.

^σ^ Based on the largest diameter of the nodule.

^ν^ Statistical difference was found in solid and ground glass opacity between the female and male.

### Guideline-based characteristics of Lung-RADS 4 participants

Among the 75 participants with Lung-RADS 4 lung nodules, the vast majority were older than 50 years (72.0%), with the mean and standard deviation of age being 54.3 and 10.2 years. Nearly all women with Lung-RADS 4 were non-smokers (97.3%), while men with Lung-RADS 4 showed an equal proportion of smokers and non-smokers (42.1%), with the remaining 15.8% being ex-smokers. Only a few of the Lung-RADS 4 participants had passive smoking history (13.8%) and a family history of lung cancer (8.0%). It is worth noting that related information was missing among the 42 (72.4%) participants with Lung-RADS 4 lung nodules for passive smoking history. Around nine-tenth (90.7%) of the participants with Lung-RADS 4 lung nodules had no hazardous occupation exposure. The vast majority of participants followed guideline recommendations for LDCT screening (88.0%). However, nearly a quarter of women (24.3%) did not meet any of the high-risk factors in the guidelines, compared with male who totally followed the guidelines (**Table 5**).

**Table 5:** Guideline-based characteristics of Lung-RADS 4 participants^α^.

Characteristics	Total (N=75)	Female (N=37)	Male (N=38)	*p* value
Lung-RADS ^ρ^ category				0.495
Lung-RADS 4A	29 (38.7)	13 (35.1)	16 (42.1)	
Lung-RADS 4B	40 (53.3)	22 (59.5)	18 (47.4)	
Lung-RADS 4X	6 (8.0)	2 (5.4)	4 (10.5)	
Age range (years)				
≤30	3 (4.0)	1 (2.7)	2 (5.3)	
31-40	4 (5.3)	2 (5.4)	2 (5.3)	
41-50	14 (18.7)	11 (29.7)	3 (7.9)	
51-60	37 (49.3)	18 (48.7)	19 (50.0)	
61-70	14 (18.7)	5 (13.5)	9 (23.7)	
≥71	3 (4.0)	0 (0.0)	3 (7.8)	
**Mean age ± SD** ^δ^ **(years)**	54.3±10.2	52.3±8.7	56.2±11.1	0.452
Smoking status				<0.001^τ^
Non-smokers^β^	52 (69.3)	36 (97.3)	16 (42.1)	
Smokers	17 (22.7)	1 (2.7)	16 (42.1)	
Ex-smokers	6 (8.0)	0 (0.0)	6 (15.8)	
Passive smoking history ^ζ^				0.031
Yes	8 (13.8)	4 (11.1)	4 (18.2)	
No	8 (13.8)	2 (5.6)	6 (27.3)	
Unknown	42 (72.4)	30 (83.3)	12 (54.5)	
Family history of lung cancer ^θ^				0.565
Yes	6 (8.0)	2 (5.4)	4 (10.5)	
No	57 (76.0)	30 (81.1)	27 (71.1)	
Unknown	12 (16.0)	5 (13.5)	7 (18.4)	
Hazardous occupation ^μ^				>0.999
Metal smelting	2 (2.7)	1 (2.7)	1 (2.6)	
Cook	4 (5.3)	2 (5.4)	2 (5.3)	
Related to pesticide exposure	1 (1.3)	0 (0.0)	1 (2.6)	
None	68 (90.7)	34 (91.9)	34 (89.5)	
Guideline compliance				0.001^τ^
Yes	66 (88.0)	28 (75.7)	38 (100.0)	
No	9 (12.0)	9 (24.3)	0 (0.0)	

^α^ The values are numbers (percentages), unless stated otherwise.

^ρ^ Lung-RADS means Lung Imaging Reporting and Data System.

^δ^ SD means Standard Deviation.

^τ^ Statistical difference was found between the female and male.

^β^ Non-smokers were defined as never smoked or had smoked fewer than 100 cigarettes in their lifetime.

^θ^ Family history limited to first-degree relatives.

^μ^ Exposed to occupational hazardous substances for one year or more.

## Discussion

In this study, we summarized the screening guideline–based characteristics and the detected lung nodule characteristics of 5,486 participants who received LDCT screening in a tertiary teaching hospital in Hunan, China in the year 2021. Interesting findings are yielded: (1) over one-quarter of the participants who received screening did not meet the criteria of the guideline-defined high-risk population, with non-smokers showing an even higher percentage (36.4%); (2) although many participants were found to have lung nodules, no clinical intervention was required basically; (3) among non-smoking women, ground glass opacity appeared to be more common; (4) there might be additional risk factors to be explored in Lung-RADS 4 participants, especially for female non-smokers as around one-quarter of them did not have any guideline-defined high-risk factors. Improvements are needed for a better criterion of participants for lung cancer screening as well as more precise definition for the high-risk population.

Early screening has considered to be an effective way to reduce lung cancer mortality and improve patient prognosis (34). Over the past 40 years, LDCT has been used for lung cancer screening among heavy smokers and has demonstrated a promising effect (33). Guidelines have been issued to promote lung cancer screening among high-risk individuals (13–15, 35), who would be most likely to benefit from the screening with less harm (e.g., overscreening and overdiagnosis). It is necessary to investigate whether the participants who received the LDCT screening meet the criteria of high risk defined by the guideline. Our study indicated that many participants (74.0%) did match the definition of the target population for screening in the latest Chinese guidelines (15) based on a sample of participants from a tertiary hospital in China. However, there were still a quarter of the participants who underwent the LDCT screening who did not meet the guideline-defined high-risk populations. Whether these participants could benefit from the screening needs further evidence.

*The commonly seen physical examinations in hospital settings in China arouse the concerns about over-screening (* 36)*. Overscreening might lead to physical impairment* (e.g., overly invasive diagnosis (37, 38) and overtreatment (39, 40)) *and psychological stress (e.g., anxiousness and insomnia) to the participants. There is no doubt that doctors (different decision-making attitudes) and medical institutions (different lung nodule management and follow-up plans) play important roles in appropriate lung cancer screening. Participants’ knowledge and attitude might also impact the screening decision. It is necessary to conduct further surveys to explore the participants’ knowledge of LDCT screening (including benefits and side effects) and the reasons for receiving screening, especially those who do not meet the definition of a high-risk population. Furthermore, Chinese screening guidelines (* 15) *recommend implementing a risk assessment of all individuals who come for LDCT screening to identify the target population. That could reduce unnecessary screening to some extent if implemented well.*

Another important issue regarding LDCT screening is that there is no standardized definition for positive nodules. Different thresholds showed large variation in positive results, which are directly related to the subsequent re-examinations and the patient’s psychological state (12). The NLST was based on the definition of positive nodules with non-calcified nodules ≥4 mm along with a detection rate of 24.2% (28, 37). In a lung cancer screening study focusing on non-smoking populations in South Korea from Asians (25), positive nodules were defined as any non-calcified nodule larger than 3 mm in diameter while the positive rate of nodule screening was only 10.0%. One previous study (29) focusing on the initial LDCT physical examination data of Shanghai residents suggested a size of 5 mm as the threshold for positive results to reduce the negative effects of screening with a 29.9% nodule detection rate. In our study, although the detection rate of nodules was higher than mentioned above (differences in machine equipment or parameter settings (41, 42) might be important reasons), most nodules were Lung-RADS 2, which were relatively safe and did not require clinical treatment (15). Furthermore, individuals might react very differently to the screening results. If all were informed as positive, some of the screen-positive people who are too anxious and worried to wait for follow-up (29) might urgently seek surgical intervention, which may be considered unnecessary (43). That causes clinicians to have a dilemma of how to inform participants about the screening results. Subsequent studies are needed to explore individualized definitions of positive nodules in combination with treatment regimens, patient psychological status, patient risk factors for lung cancer, and other risk indicators for nodules.

The 10-year follow-up results from the Nederlands–Leuvens Longkanker Screenings Onderzoek (NELSON) indicated that lung cancer screening is more beneficial for women than men (RR: 0.67, 95%CI: 0.38–1.14). Our results indicated that the proportion of ground glass opacity was higher in non-smoking women, which was consistent with the results of previous studies (44, 45). Current studies suggest that ground glass opacity progresses slowly, but its malignancy is higher than that of solid nodules (46). Given that East Asian women are more likely to develop non-smoking lung cancer (47), more attention should be paid to the non-smoking female population with ground glass opacity, especially in follow-up and nodule management after the initial screening.

Among the participants with Lung-RADS 4 that were considered to have the strongest potential link to lung cancer, no guideline-defined high-risk factors still existed, especially among women, with one in four having no guideline-defined high-risk factors. Missing data and missing variables not covered in the pre-examination questionnaire (e.g., a history of COPD and passive smoking) might contribute. However, it can still be considered that the current definition of high-risk groups has the value of continuing to explore. Refinement on the criteria of high risk might also be considered by taking account of updated and localized evidence. An increasing number of young people and non-smoking female have been diagnosed with lung cancer, are gradually changing (45, 48). Additional inconclusive risk factors for lung cancer should be consistently explored through high-quality studies in order to uniquely guide different populations. Considering the continuous emergence of new evidence, while fully pondering the cost-effectiveness (49), relevant screening guidelines should also be updated in a timely manner.

There are some limitations in our study. Firstly, all included participants in our study were from one hospital in Hunan. This study was based on data from a single center and was cross-sectional in nature, which limited the generalizability of the results from this study. Regional and ethnic differences should also be taken into account when extrapolating the conclusions.

Secondly, guideline-based characteristics were derived from the self-administrated pre-examination questionnaires, which might be accompanied by information bias. Therefore, when duplicate questionnaires were found for the same person, we chose the most comprehensive one to compensate bias. As participants are explained that the pre-examination questionnaires will facilitate a better accurate diagnosis from the doctor, we are confident that the information in our study should have reflected part of current screening participants’ situations.

Thirdly, our study excluded those who participated in lung cancer screening but missing the pre-examination questionnaire information, which may be accompanied by selection bias. However, even based on a relatively small sample, results from this study indicate an unsatisfactory guideline adherence of LDCT screening at a hospital setting. Clinicians are suggested to pay attention to the criteria of the guideline while prescribing LDCT for patients. Follow-up data from multicenters are needed to ensure a better external validity in this research topic. Lung cancer screening guidelines need to be widely promoted to reduce over-screening and promote better allocation of medical resources.

Lastly, with the results of the initial LDCT screening in our study that only provided baseline information, the prognosis of the participants with or without different screening results is unknown. Subsequent studies are needed to follow up the same population to investigate the prognosis as well as prognostic factors to inform a better applicable nodule management strategy.

## Conclusion

Based on data from a hospital in Hunan, China, most individuals who received LDCT screening for lung cancer did meet the guideline-defined high-risk population criteria; however, over one-quarter of the participants who received screening did not meet the criteria of the guideline-defined high-risk population, especially among non-smokers. The positive rate varied largely according to different cut-off values for positive nodules. Appropriate cut-off values for positive nodules need to be continuously explored; further, more precise and localized criteria for high-risk individuals are needed, and more attention should be given to non-smoking women in lung cancer screening.

## Data availability statement

The raw data supporting the conclusions of this article will be made available by the authors, without undue reservation.

## Ethics statement

The studies involving human participants were reviewed and approved by the Ethics Committee of Xiangya School of Public Health, Central South University (XYGW-2021-104). The patients/participants provided their written informed consent to participate in this study.

## Author contributions

ZZ and IW formulated the theme. SL and BD collected the data. YG and ZZ finished statistical analysis. ZZ and XZ wrote the main manuscript text and prepared all figures and tables. All authors contributed to the article and approved the submitted version.
